# Associations of trait emotional intelligence and stress with anxiety in Chinese medical students

**DOI:** 10.1371/journal.pone.0273950

**Published:** 2022-09-01

**Authors:** Meng Shi, XiaoShi Lu, TianJiao Du

**Affiliations:** 1 Department of Linguistics, Institute of Foreign Languages, China Medical University, Shenyang, P.R. China; 2 Department of Psychology, School of Medical Humanities, China Medical University, Shenyang, P.R. China; 3 The 3rd Clinical Department, China Medical University, Shenyang, P.R. China; Universidad Austral de Chile, CHILE

## Abstract

**Background:**

Medical students are vulnerable to anxiety. Identifying its key influencing factors can potentially benefit both students and their future patients. Trait emotional intelligence (EI) and perceived stress may play important roles in anxiety. The main objective of this study was to examine the associations between trait EI, perceived stress and anxiety among Chinese medical students.

**Materials and methods:**

Self-report questionnaires, consisting of the Chinese versions of the Trait Emotional Intelligence Questionnaire-Short Form, the 10-item Perceived Stress Scale, the 7-item Generalized Anxiety Disorder Scale, and demographic section were distributed to 1500 students at three medical universities in China. Hierarchical regression analyses were performed to examine the associations between trait EI, perceived stress and anxiety. Asymptotic and resampling strategies were utilized to examine the mediating effect of perceived stress.

**Results:**

A total number of 1388 medical students became final participants. After adjustment for demographics, while trait EI was strongly and negatively associated with anxiety, accounting for 21.1% of its variance, perceived stress was strongly and positively related to anxiety, explaining an additional 10.0% of the variance. Stress appeared to have a mediating effect on the association between trait EI and anxiety in students with high and moderate levels of stress, but not in low stress group.

**Conclusions:**

Both constructs of trait EI and perceived stress could be of vital importance to understand anxiety in medical students. Evidence-based strategies to enhance trait EI and reduce perceived stress might be undertaken to prevent and treat anxiety in the students.

## Introduction

A recent meta-analysis suggests that approximately one-third of medical students have been found to suffer from anxiety [1]. Anxiety has become the sixth leading cause of disability worldwide, with its highest burden of disease for people aged between 15 and 34 [2]. In addition to persistent and excessive fear or worry that significantly interferes with daily life, anxiety sufferers can experience a series of physiological symptoms such as headache, fatigue, and nausea [3]. Anxiety in medical students warrants more attention, given its high prevalence, heavy disease burden, and negative impacts on academic performance, quality of life, professionalism, and patient care quality [1, 2]. Research has suggested that development of anxiety is largely attributed to non-genetic factors [4]. Thus, identifying its key influencing factors in medical students is of vital significance to develop effective intervention strategies. Emotional intelligence (EI) underpins many of the skills that contribute towards the core competencies of medical students, and is essential to high quality patient care and can benefit both students and their future patients [5].

Empirical studies have shown that EI may be an important predictor of anxiety [6]. It fundamentally refers to the competence to identify, express, understand and regulate emotions in the self and in others [7]. These intrapersonal and interpersonal competencies of EI can facilitate adaptation and may play pivotal roles in anxiety [8]. The conceptualization of EI can be dichotomized into trait EI and ability EI. While trait EI is defined as a constellation of emotional perceptions assessed through questionnaires and rating scales, ability EI concerns emotion-related cognitive abilities measured via performance-based tests [9]. In the current study, the trait EI model was adopted as it was revealed to have solid theoretical foundation [10] and provide the most appropriate framework within which to investigate the general emotional functioning of people [11]. Using different measures, some recent studies render consistent evidence showing that trait EI is negatively associated with anxiety in both healthy and clinical populations [12, 13], which is congruent with the findings of the comprehensive meta-analysis regarding the relation between trait EI and mental health [6]. As-of-yet, however, there are few studies investigating the relation between trait EI and anxiety in medical students, and these few studies were carried out almost exclusively in Western cultures and usually consisted of relatively small samples [14, 15]. In addition, though psychometric properties of trait EI measures have been confirmed across cultures [16], significant difference in trait EI has been revealed between individualist cultures and collectivist ones [17]. It is worth exploring the relationship between the two constructs in different cultural contexts. Thus, using a large multi-site sample, we conducted this study to examine the association of trait EI with anxiety in Chinese medical students.

While prior studies have investigated the relationship between trait EI and anxiety, the potential mechanism underlying the relation remains largely unclear. Trait EI is also called trait emotional self-efficacy, which concerns self-perceptions of one’s ability to recognize, process, and utilize emotion-laden information [18]. According to the theory of self-efficacy, people with a stronger sense of perceived self-efficacy can reduce stress and maintain psychological health better [19]. Many empirical studies have demonstrated negative association of trait EI with stress perception [20, 21]. For instance, individuals with higher trait EI have displayed significantly lower levels of perceived chronic and occupational stress than those with lower trait EI [21]. Trait EI has been also revealed to be a negative predictor of perceived stress in medical students over time [22]. However, it is worth noting that the relation between trait EI and stress is not always consistent. Arora et al., for example, have shown that medical students with higher trait EI experience more stress during unfamiliar surgical scenarios compared with their lower-trait-EI peers [23]. In addition to perceived stress, trait EI also has been shown to be related to physiological stress response, such as heart rate variability and cortisol levels [21, 24]. Thus, both theory and empirical evidence have indicated that trait EI can play a pivotal role in stress perception.

Stress has been defined as the strain that accompanies a demand perceived to be either challenging (positive) or threatening (negative) and, depending on its appraisal, may be either adaptive or debilitating [25]. It is closely linked to anxiety, and one debilitating reaction to stress is anxiety [25–27]. A 20-year longitudinal study of the impact of stress exposure suggests that life stress events predict anxiety in early adulthood, and a reduction in stress exposure is protective against anxiety [26]. With regard to college students, academic distress and financial stress are important predictors of their anxiety [28, 29]. The effects of stress on anxiety in epidemiologic studies have been confirmed by neurobiological findings [30]. While both constructs of stress and anxiety have been extensively studied in medical students, the relationship between stress and anxiety has rarely been examined in this population. The exploration of this relationship may be conducive to developing intervention strategies to relieve psychological distress in medical students. Meanwhile, though the associations of stress with trait EI and anxiety have been examined respectively, the possible mediating role that stress may play in the relationship between trait EI and anxiety has yet to be explored. Thus, we conducted the present study with the following objectives: 1) to investigate the associations between trait EI, stress and anxiety in Chinese medical students; 2) and to examine the mediating effect of stress on the relationship between trait EI and anxiety.

## Materials and methods

### Participants

This multicenter cross-sectional study was conducted at three medical schools in China, including China Medical University, Xiangya School of Medicine, and Guizhou Medical University. The dominant model of medical education in China is 5-year undergraduate program, and students of the first four years were chosen as participants. Based on study year, stratified random cluster sampling was used to select whole classes of students from each institution. 1500 self-report questionnaires were distributed and 1396 were returned. A pool of 1388 students (effective response rate: 92.5%) became the final participants after eight invalid questionnaires were excluded, and missing values were replaced with mean substitution approach. The study was approved by the Institutional Review Board of China Medical University, and written informed consent was obtained from each participant according to the Declaration of Helsinki.

### Measures

#### Measurement of anxiety

Anxiety was measured with the 7-item Generalized Anxiety Disorder Scale (GAD-7) [31]. Each item (e.g., “Over the last 2 weeks, how often have you been bothered by the following problems? Not being able to stop or control worrying?”) is rated on a 4-point Likert scale according to the frequency of the symptoms during the last 2 weeks, ranging from 0 (not at all) to 3 (nearly every day). Higher overall scores indicate higher levels of anxiety, with scores of 5, 10, and 15 representing mild, moderate, and severe anxiety, respectively [31]. The cutoff score of ≥ 10 was adopted in the study. The Chinese version of the GAD-7 has demonstrated adequate reliability and validity [32], and in the current study, its Cronbach’s alpha coefficient was 0.92.

#### Measurement of trait EI

Trait EI was measured using the Trait Emotional Intelligence Questionnaire-Short Form (TEIQue-SF), which consisted of 30 items (e.g., “I’m usually able to find ways to control my emotions when I want to”) [33]. Each item is scored on a 7-point Likert scale from 1 (completely disagree) to 7 (completely agree), with higher scores indicating higher levels of self-reported trait EI. The Chinese version of the TEIQue-SF has shown adequate psychometric properties [34]. In the study, we only used the global trait EI score and not the factor-level as the TEIQue-SF is primarily intended to measure global trait EI, although factor scores, which tend to have lower internal consistencies, can be derived [33–35]. The Cronbach’s alpha for global trait EI was 0.91.

#### Measurement of stress

The 10-item Perceived Stress Scale (PSS) was used to measure the perception of stress in the students [36]. Items of the PSS (e.g., “In the last month, how often have you found that you could not cope with all the things that you had to do?”) are scored on a 5-point Likert scale, ranging from 0 (never) to 4 (very often). Higher scores indicate higher levels of perceived stress. The Chinese version of the PSS has demonstrated adequate reliability and validity [37, 38], and the Cronbach’s alpha was 0.86 in the study.

#### Demographic characteristics

Demographic data regarding age, gender, study year, and hometowns were obtained. Hometowns were dichotomized into urban and non-urban areas.

### Statistical analysis

Statistical analyses were performed using SPSS 25.0. All statistical tests were two-sided and the significance level was set at *p* < 0.05. Independent sample t-tests, one-way ANOVA and Bonferroni post hoc tests were performed to compare students’ differences of anxiety in categorical variables. Pearson’s correlation was used to examine correlations among the continuous variables. Hierarchical regression analyses were conducted to examine the associations between trait EI, perceived stress and anxiety. In step 1, the demographic variables were entered as control variables; in step 2, trait EI was entered; in step 3, perceived stress was added; in step 4, the product of trait EI and perceived stress was entered. If the interaction effect was statistically significant, we would investigate the mediation effects in simple slope analyses. Asymptotic and resampling strategies were used to examine the mediating effect of stress on the association of trait EI with anxiety. Bias-corrected and accelerated 95% confidence interval (BCa 95% CI) for the a * b product was calculated based on 5000 bootstrap samples, and a BCa 95% CI excluding 0 indicated a significant mediating effect [39].

## Results

### Characteristics of participants and prevalence of anxiety

Demographics of the medical students and distributions of anxiety in categorical variables are shown in Table 1. Of 1388 students, 591 (42.6%) were males, and 797 (57.4%) were females. Their ages ranged from 17 to 25 (M = 19.79, SD = 1.50). 703 students (50.6%) came from cities, whereas 685 (49.4%) were from non-urban areas. The prevalence of anxiety among the students was 9.5% (GAD-7 ≥ 10). Compared with first year students, students of later stage showed higher levels of anxiety (*p* < 0.001). Students from non-urban areas showed higher level of anxiety than those from urban areas (*p* < 0.001).

**Table 1:** Demographic characteristics and differences in anxiety (N = 1388).

Variables	N	%	GAD-7 (Mean ± SD)	t/F	P	Cohen’s d
Gender						
Male	591	42.6%	4.31 ± 4.09	1.913	0.056	0.10
Female	797	57.4%	3.89 ± 3.95			
Study year						
First year	440	31.7%	3.30 ±3.70^a^	9.007	< 0.001	0.33
Second year	338	24.4%	4.11 ± 3.95^b^			
Third year	303	21.8%	4.64 ± 4.07^b^			
Fourth year	307	22.1%	4.54 ± 4.29^b^			
Hometowns						
Urban area	703	50.6%	3.68 ± 3.89	3.652	< 0.001	0.20
Non-urban area	685	49.4%	4.46 ± 4.11			

GAD-7: the 7-item Generalized Anxiety Disorder Scale

Different lower-case superscript letters indicate significant differences between the groups (*p* < 0.05)

### Correlations among study variables

Means, standard deviations of the continuous variables and the results of Pearson correlation analyses are presented in Table 2. Age was negatively related to trait EI (*r* = −0.12, *p* < 0.01), but positively related to stress (*r* = 0.07, *p* < 0.01) and anxiety (*r* = 0.14, *p* < 0.01). Trait EI was negatively correlated with stress (*r* = −0.65, *p* < 0.01) and anxiety (*r* = −0.48, *p* < 0.01). Stress was positively related to anxiety (*r* = 0.55, *p* < 0.01).

**Table 2:** Means, standard deviations (SD) and correlations of continuous variables.

Variables	Mean	SD	1	2	3	4
1. Age	19.79	1.50	1			
2. Trait EI	140.51	21.51	−0.12**	1		
3. Stress	15.42	5.93	0.07**	−0.65**	1	
4. Anxiety	4.06	4.01	0.14**	−0.48**	0.55**	1

**p < 0.01 (two-tailed)

### Hierarchical regression analyses

Results of the hierarchical regression analyses are shown in Table 3. Multicollinearity between predictors, including trait EI, factors of trait EI, and stress, was all inspected.Values for the variance inflation factor (VIF) ranged from 1.66 to 2.44, indicating no concern for multicollinearity. While the demographic factors explained only a small proportion of its variance, trait EI had a strong negative association with anxiety (*β* = −0.467, *p* < 0.01). Perceived stress was shown to have a strong positive association with anxiety (*β* = 0.415, *p* < 0.01). The interaction term between trait EI and stress was also significantly associated with anxiety (*β* = −0.155, *p* < 0.01), accounting for an additional variance above and beyond what was explained by the covariates and main effects.

**Table 3:** Hierarchical linear regression analyses results.

Variables	Block 1 (*β*)	Block 2 *(β*)	Block 3 (*β*)	Block 4 (*β*)
Age	0.066	0.049	0.043	0.046
Gender	− 0.048	−0.024	−0.031	−0.031
Hometowns	0.074**	0.018	0.030	0.038
Grade dummy 1	0.061	0.030	0.012	0.014
Grade dummy 2	0.096*	0.066	0.061	0.055
Grade dummy 3	0.063	0.032	0.037	0.033
Trait EI		−0.467**	−0.197**	−0.225**
Stress			0.415**	0.418**
Trait EI × Stress				−0.155**
F	7.252**	62.824**	89.317**	87.692**
R^2^	0.031	0.242	0.341	0.364
△R^2^		0.211	0.100	0.023

**p < 0.01 (two-tailed)

*p < 0.05 (two-tailed)

Grade dummy 1: year 2/year 1; Grade dummy 2: year 3/year 1; Grade dummy 3: year 4/year 1

### Mediating effect of stress on the relationship between trait EI and anxiety

**Fig 1:**
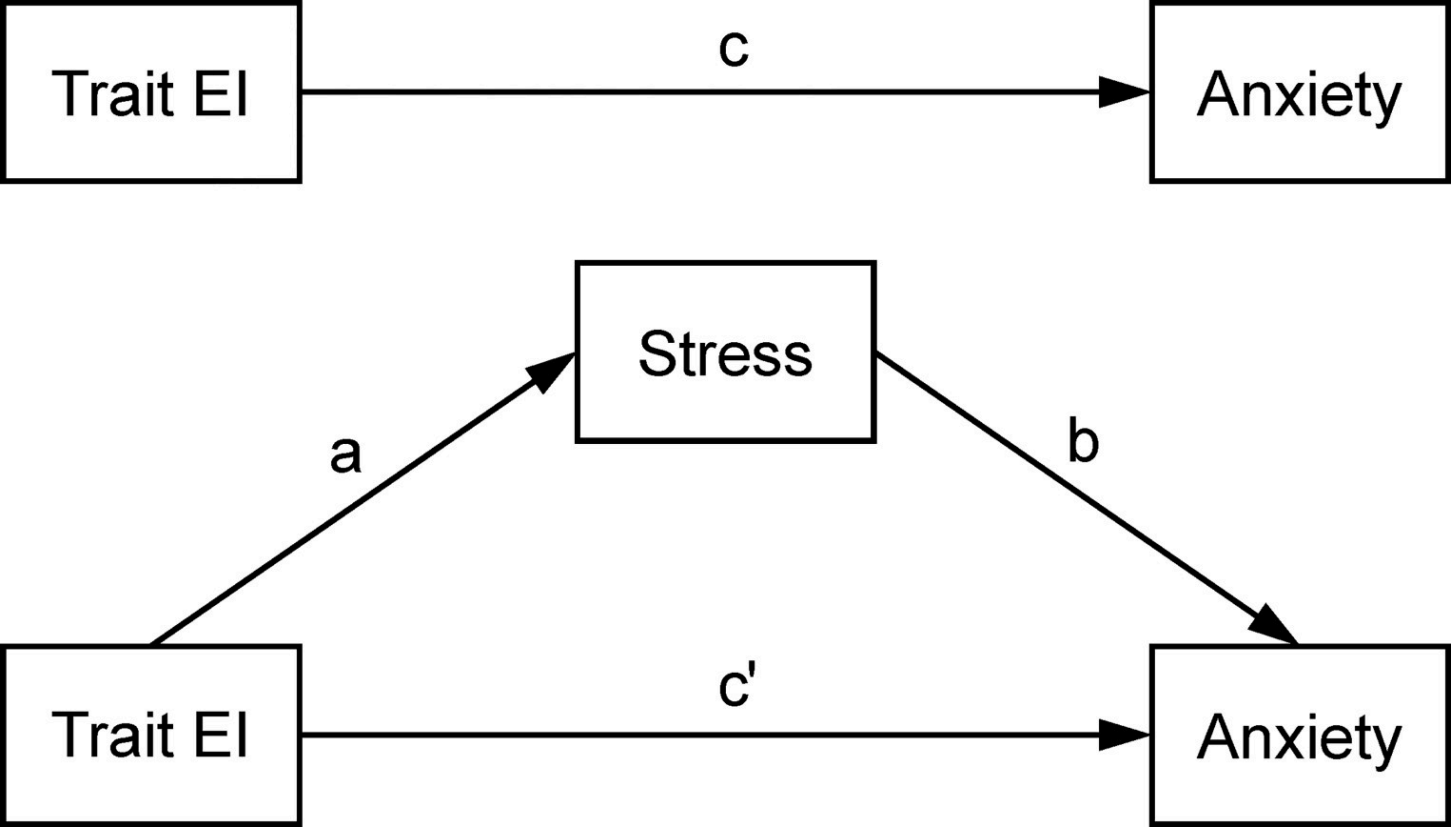
Mediation model of stress on the association of trait EI with anxiety. *a*: the association of trait EI with stress; b: the association of stress with anxiety after controlling for the predictor variable; c: the total effect of trait EI on anxiety; c′: the direct effect of trait EI on anxiety after adding stress as a mediator. c = ab+c′.

Results of the asymptotic and resampling strategies showed that trait EI was negatively associated with stress (*β* = −0.649, *p* < 0.001), and stress was positively associated with anxiety (*β* = 0.416, *p* < 0.001). Stress mediated the association of trait EI with anxiety with an effect size of −0.050 (BCa 95% CI: −0.059, −0.042). The proportion of the mediation effect was 57.47% in the total effect of trait EI on anxiety. We then conducted three mediation analyses in high (> 1SD above the mean, n = 185), moderate (within mean ± 1SD, n = 992), and low (< 1SD below the mean, n = 211) stress groups, respectively. The mediation effect size was −0.024 (BCa 95% CI: −0.048, −0.006) for the high stress group, −0.025 (BCa 95% CI: −0.032, −0.018) for the moderate stress group, and −0.003 (BCa 95% CI: −0.010, 0.004) for the low stress group, which was statistically non-significant. The mediation model is presented in Fig 1.

## Discussion

This large multicenter study examined the associations between trait EI, perceived stress and anxiety among Chinese medical students. The results showed that trait EI was negatively associated with anxiety, which confirmed the findings of prior studies conducted in Western cultures and in other population groups [12–15]. High trait EI individuals are more capable of regulating their emotions in a flexible and adaptive manner and taking effective coping strategies than their counterparts, both of which are conducive to reducing anxiety. For instance, confronted with a negative situation, high trait EI people are more likely to strive to change the situation via different modification strategies; when the situation cannot be changed, they are more prone to use reappraisal and acceptance strategies [40]. Also, they are more able to utilize emotional skills to repair negative moods to facilitate emotional recovery. Though they could be more sensitive, people with high trait EI possess greater ability to put emotions aside when it is necessary [41].

A major finding of the study is that it sheds some light on the role of perceived stress on the relationship between trait EI and anxiety. Perceived stress was found to mediate the association of trait EI with anxiety among medical students with high and moderate levels of stress. Lower level of trait EI was associated with higher stress, which was in turn correlated with higher level of anxiety. According to the transactional model of stress [42], trait EI can be considered a crucial personal resource affecting appraisals of environment and coping strategies. Individuals with low trait EI lack self-efficacy in coping with stress and view stressful situations as more of a threat than a challenge [43]. The positive link between trait EI and interpersonal relationships can also prevent low trait EI people from navigating daily stressors in various social contexts [44]. Meanwhile, high level of stress has consistently been shown to predispose individuals to anxiety [26, 27]. The mediation effect was not statistically significant in the low stress group. One possible explanation is that people with low stress may possess more coping resources to manage challenges in daily life and use adaptive coping more often, both of which can reduce the indirect effect of trait EI on anxiety through stress [45].

The findings of the present study may have significant implications for medical education, particularly in Chinese context. Medical students in China may be more susceptible to high levels of anxiety and stress than their counterparts in many other nations due to the following factors. In addition to heavy workload, physicians in China have to face serious challenges, such as patient-physician mistrust, violence against physicians [46], which can negatively affect mental health of Chinese medical students. In order to land jobs in cities, Chinese undergraduate medical students have to prepare for graduate school entrance examination years ahead to pursue advanced degrees, which can also significantly increase their psychological distress. However, rather low percentage of distressed medical students use mental health counseling services due to its related stigma and concerns about their future careers [47]. Anxiety in medical students, if left untreated, may extend into physician stage, affecting both wellbeing of students and quality of health care. Thus, effective intervention strategies to prevent and treat anxiety in medical students are of vital significance. The results of this study indicate that trait EI could be an important predictor of anxiety. According to the interactional model and life span model of personality development, personality traits do change across the life course, and the changes can be substantial during the period of young adulthood [48]. Empirical evidence has shown that trait EI is malleable. Both a recent meta-analysis and a systematic review have provided substantial support for the efficacy of trait EI interventions in adults [49, 50]. In a study, for instance, adult participants of an intervention group attended a two-and-a-half day training on emotional competencies. After the intervention, compared with non-significant change in control group, the level of trait EI in intervention group significantly increased, with additional benefits for other psychological, somatic, and social indicators. The changes were consistent between self-report and informant measures, and maintained one year later [51].

In the study, our data suggest that perceived stress may exert a mediating effect on the association of trait EI with anxiety in the students with high and moderate levels of stress. Thus, stress management programs warrant more attention to help medical students better cope with anxiety. Certain evidence-based intervention strategies have been demonstrated to be effective in reducing stress. For example, meta-analyses have revealed that mindfulness-based stress reduction (MBSR) can help individuals significantly lower their stress [52, 53]. Heart rate variability (HRV) biofeedback is another intervention that has gained increasing attention and showed promising effects in alleviating psychological stress in diverse groups of people [54, 55]. It is particularly noteworthy that both interventions, MBSR and HRV biofeedback, are cost- and time-effective, have no side effects, and involve minimal stigma [27], thus have great potential to be used as standard solutions for medical students to deal with stress in their daily life.

Some limitations of the study should be acknowledged. First, causal relations among the study variables cannot be drawn due to the cross-sectional design. The findings should be confirmed by prospective cohort studies in the future. Second, all data were obtained through self-report questionnaires, which could introduce response bias. Third, other factors, such as coping, were not considered potential mediators between trait EI and anxiety, and could be investigated in the future, especially for low stress people. Fourth, the generalization of the results should be taken with caution. More studies should be conducted in other cultural contexts.

## Conclusions

This study revealed that trait EI could be a vital protective factor against anxiety in Chinese medical students, and perceived stress appeared to have a mediating effect on the association of trait EI with anxiety in students with high and moderate levels of stress. Thus, evidence-based intervention strategies to cultivate trait EI and reduce perceived stress might be undertaken to prevent and treat anxiety in medical students.

## Supporting information

S1 Dataset (SAV)
